# Bilateral Vaginocutaneous Fistulas Three Years following the Insertion of a “Tension-Free” Vaginal Tape

**DOI:** 10.1155/2012/231824

**Published:** 2012-12-26

**Authors:** James Bacon, Penny Black

**Affiliations:** ^1^Department of Obstetrics and Gynaecology, Royal North Shore Hospital, Reserve Road, St Leonards, Sydney, NSW 2065, Australia; ^2^Port Macquarie Base Hospital, Suite 6, 2 Wrights Lane, Port Macquarie, NSW 2444, Australia

## Abstract

We present a rare complication of fistula following the insertion of a transvaginal tape and the literature surrounding fistula formation following this procedure.

## 1. Introduction

The aim is to present a case of bilateral vaginocutaneous fistulas following the insertion of a polypropylene tension-free vaginal tape (TVT) for the treatment of genuine stress incontinence. This is a rare complication, as demonstrated by a literature review of TVT complications, and is relevant in the setting of recent FDA reports on mesh safety. This will be followed by a discussion of the treatment of vaginal fistulas and erosions caused by the insertion of TVT.

## 2. Case Report

 A 42-year-old woman presented to clinic with persistent vaginal discharge, dyspareunia, and postcoital bleeding. A polypropylene tension-free vaginal tape (TVT) had been inserted 40 months prior for genuine stress incontinence. Her background history included a total abdominal hysterectomy eight years previously for menorrhagia, two normal vaginal deliveries, and one elective lower uterine segment caesarean section for a breech presentation. The patient had no comorbidities.

 Her symptoms began with painless postcoital bleeding 4 months after the TVT was inserted, which was extensively investigated and no cause was found. This was followed by persistent vaginal discharge, pruritus and dyspareunia. Again, following investigation no cause was found in particular swab cultures negative however, there was symptomatic improvement with multiple courses of antibiotics.

 Almost three years later, with worsening discharge and dyspareunia, the patient was seen in clinic and booked for a diagnostic laparoscopy. She presented one week prior to surgery with a discharging cellulitic pubic abscess; a fistulogram demonstrated a left-sided vaginocutaneous fistula with no peritoneal communication. Vaginal and abdominal approaches were used to remove the tape from both the left-sided abscess and the right side however, not all the tape could successfully be removed from the right side. Six months later the patient returned to clinic with a right-sided discharging suprapubic abscess, which was diagnosed clinically as an infected vaginocutaneous fistula. A three-week course of oral antibiotics did not resolve the symptoms, so the patient underwent surgical removal of the remaining tape followed by 48 hours of intravenous antibiotics. At six weeks followup the patient's abdominal wall lesion was healing and she was free from vaginal discharge and bleeding. The patient's main concern was the reoccurrence of her stress incontinence.

## 3. Discussion

Urinary incontinence affects between 25 and 45% of the female population, with stress incontinence being recognized as the most common form. In 1996 the tension-free vaginal tape procedure was described that involved placing a synthetic mesh (polypropylene) under the midurethra to alleviate the symptoms of stress incontinence. This tape was hypothesized to increase the vaginal hammock supports and assist in the activity of the pubourethral ligaments and levator animuscles [1]. An 11-year followup of TVT insertions demonstrated a 90% cure rate for stress incontinence on both qualitative and quantitative measures [2]. 

 Multiple complications of this procedure have been documented, including intraoperative bleeding, hematoma formation, postoperative voiding dysfunction, tape erosion, recurrent urinary tract infections, and wound infection [3]. In July 2011, the FDA released a statement regarding the potential complications associated with mesh in particular erosion and pain, over a period between 2008 and 2011; 1371 complications were reported in stress incontinence operations [4].

The causes of these complications are multifactorial with operator experience, material selection, and patient selection playing a crucial role. Tapes that are placed under too much tension can cause tissue ischemia and necrosis. Materials should be nontoxic and nonantigenic, with grafts containing pores larger than 75 microns (such as polypropylene) resulting in reduced rates of infection [5].

 We conducted a comprehensive literature review of cases of vaginocutaneous fistulas following transvaginal tape insertion and their subsequent treatment. Electronic searches of literature published between January 1995 and May 2010 were undertaken using Medline, EMBASE, Pubmed, and the Cochrane Database of Systematic Reviews. Search terms included “tension-free vaginal tape,” “transvaginal tape,” or “TVT” combined with the terms “fistula,” “infection,” or “complication.” Results were limited to the English language.

 The literature on vaginocutaneous fistula formation is sparse with no overall incidence published. A systematic review and meta-analysis failed to recognize any cases of delayed cutaneous infection following TVT insertion [6]. There have been two long-term follow-up studies of tension-free vaginal tapes over 11 years involving a total of 237 women with no reports of any long-term adverse outcomes, in particular no vaginocutaneous fistulas or abscesses [2, 7].

There are only three reports of similar case presentations of delayed infection following synthetic tape insertion in the literature. One report is of a vaginocutaneous fistula 18 months following bladder neck suspension, the Stamey cystourethropexy procedure [8]. This was managed with a removal of the nylon and pledget. This case differs from ours in that the synthetic material used has different microbiological properties to polypropylene tape, and the procedure is different. Another case is of a retropubic abscess formation 10 months post-TVT insertion requiring tape removal after failed medical therapy [9]. The patient had no ongoing problems of incontinence following tape removal. The synthetic material used was the same as our case, but our case differs in the much later timing of the complication, the formation of a vaginocutaneous fistula, and the ongoing issue of incontinence. The final report is an enterocutaneous fistula following unrecognized bowel injury at the time of insertion. This case differs from ours in regards to that there was a definite preceding incident causing the fistula formation, in this case the unrecognized bowel perforation [10].

 There have been other case reports of groin and obturator abscesses following the insertion of a trans obturator tape (a different surgical approach to TVT but using similar materials), with one report having a delay of 33 months between insertion and presentation requiring surgical removal [11]. In our case, multiple antibiotic regimes were ineffective, necessitating the surgical removal of the tape to ultimately improve the infective symptoms. Tape removal may also be required for vaginal erosions arising as a complication of TVT insertion [12].

 This particular case is unusual—the long delay in the fistula formation (40 months), the lack of comorbidities, and negative swab cultures—making the pathogenesis of the complication unclear. This case highlights the infection risk associated with synthetic materials years after insertion and the significant morbidity that can be resulted. Although the FDA is continuing to investigate mesh complications used for stress urinary incontinence, this highlights the potential risks and the need to discuss at length the possible complications with patients prior to insertion. It also illustrates the need for clinicians to closely follow up unusual and recurrent symptoms in patients with synthetic tapes and slings. The clinical problem now faced is how to treat the patient's ongoing problems of stress incontinence.
